# Mechanisms and Functions of Pexophagy in Mammalian Cells

**DOI:** 10.3390/cells10051094

**Published:** 2021-05-03

**Authors:** Jing Li, Wei Wang

**Affiliations:** 1 Department of Integrated Traditional Chinese and Western Medicine, Tongji Hospital, Tongji Medical College, Huazhong University of Science and Technology, Wuhan 430030, China; bianque129@163.com; 2 Department of Human Anatomy, School of Basic Medicine, Tongji Medical College, Huazhong University of Science and Technology, Wuhan 430030, China

**Keywords:** peroxisome, autophagy, mammalian, pexophagy, ubiquitin, receptor

## Abstract

Peroxisomes play essential roles in diverse cellular metabolism functions, and their dynamic homeostasis is maintained through the coordination of peroxisome biogenesis and turnover. Pexophagy, selective autophagic degradation of peroxisomes, is a major mechanism for removing damaged and/or superfluous peroxisomes. Dysregulation of pexophagy impairs the physiological functions of peroxisomes and contributes to the progression of many human diseases. However, the mechanisms and functions of pexophagy in mammalian cells remain largely unknown compared to those in yeast. This review focuses on mammalian pexophagy and aims to advance the understanding of the roles of pexophagy in human health and diseases. Increasing evidence shows that ubiquitination can serve as a signal for pexophagy, and ubiquitin-binding receptors, substrates, and E3 ligases/deubiquitinases involved in pexophagy have been described. Alternatively, pexophagy can be achieved in a ubiquitin-independent manner. We discuss the mechanisms of these ubiquitin-dependent and ubiquitin-independent pexophagy pathways and summarize several inducible conditions currently used to study pexophagy. We highlight several roles of pexophagy in human health and how its dysregulation may contribute to diseases.

## 1. Introduction

Peroxisomes are single-membrane organelles present in virtually all eukaryotic cells. In mammalian cells, they play essential roles in diverse cellular metabolism, such as β-oxidation of fatty acids, redox homeostasis, and the biosynthesis of bile acids and plasmalogens [1]. These metabolic activities are executed through enzymes located in the peroxisomal matrix. In addition, peroxisomes function as central signaling hubs for regulating redox and innate immune signaling [2,3].

Peroxisome biogenesis involves the following different steps: peroxisomal membrane assembly, import of matrix proteins, fission and division, and inheritance [4]. Two models have been proposed to explain peroxisome biogenesis. One is the classical growth and division model, in which new peroxisomes are derived from pre-existing peroxisomes. The other model is the de novo model, in which peroxisomes originate from vesicles derived from the endoplasmic reticulum (ER) and/or mitochondria [5]. A number of genes called peroxins (PEXs) are involved in the various stages of peroxisome biogenesis. Dysfunction of PEX genes causes fatal human peroxisome biogenesis disorders (PBDs). Zellweger syndrome (ZS) is a prototypic PBD with the most severe phenotype. PBDs caused by absent or incompetent peroxisomes highlight the importance of peroxisomes [6].

The estimated half-life of mammalian peroxisomes is 1.5~2 days, suggesting that peroxisome homeostasis is a dynamic process [7]. More importantly, the abundance and activity of peroxisomes can be rapidly adjusted to meet the metabolic needs induced by a changing environment. For example, the number of peroxisomes in human fibroblasts increases after enveloped virus infection, promoting the synthesis of the phospholipid plasmalogen required for virus replication [8]. Additionally, the number of peroxisomes increases dramatically when rodents are administered peroxisome proliferators and decreases rapidly after these drugs are withdrawn [9,10,11]. The dynamic homeostasis of peroxisomes under basal and inducing conditions is maintained through the coordination of peroxisome biogenesis and turnover.

Autophagy (“self-eating”) is a process that involves the degradation of cellular contents in lysosomes [12]. It is dramatically induced by various cellular stresses, such as nutrient scarcity and pathogen infection. Activation of autophagy serves as a crucial mechanism for cells to survive changing environmental conditions. Macroautophagy (hereafter referred to as autophagy) is the most well-studied form of autophagy, which delivers cytoplasmic material to lysosomes via the double-membraned compartment, termed autophagosome or phagophore. Autophagy has long been described as a nonselective process. However, many recent studies have revealed that autophagy can selectively degrade cargos, such as aggregates (aggrephagy), mitochondria (mitophagy), endoplasmic reticulum (reticulophagy/ER-phagy), pathogens (xenophagy), and peroxisomes (pexophagy). Selective autophagy allows for the efficient removal of certain substances in response to a particular trigger [13].

Three independent mechanisms have been proposed for peroxisome degradation in mammalian cells: the Lon protease system, autolysis, and pexophagy [14]. Excess matrix proteins, such as β-oxidation enzymes, can be digested through the Lon protease system. Autolysis depends on the activity of 15-lipoxygenase (15-LOX), which disrupts the peroxisomal membrane and induces the diffusion of the contents into the cytoplasm for proteolytic degradation. By comparison to autophagy-deficient mice, it is found that 70~80% of proliferated peroxisomes are degraded through pexophagy, and the remaining peroxisomes are removed by the Lon protease system and autolysis mechanisms [15,16]. A study showed that peroxisomes in cells of patients with PDB characterized by deficient peroxisomal AAA-type ATPase complex (AAA-complex) are degraded through pexophagy [17,18]. These studies suggest that pexophagy is a major pathway for removing excess or incompetent peroxisomes.

In yeast, peroxisome production is rapidly induced when cells are cultured in the presence of oleic acid or methanol as the exclusive carbon source. Many peroxisome enzymes are synthesized and transported into the matrix to catalyze the carbon metabolism. When the carbon sources are removed, superfluous peroxisomes are no longer needed and subjected to pexophagy. This feature has made yeast an ideal model to study pexophagy. Indeed, the mechanisms and functions of pexophagy are well elucidated in yeast and have been discussed in many excellent reviews [19,20,21,22]. However, pexophagy is more complex in mammalian cells, and studies are still in the early stages. We focus here on recent advances in our understanding of pexophagy in mammalian cells.

## 2. Ubiquitin-Dependent Pexophagy

Autophagic selectivity largely relies on unique receptors, most of which consist of a ubiquitin-binding domain (UBD) and an LC3-interacting region (LIR). These receptors act as mediators that recognize the marked ubiquitinated cargos through the UBD and deliver them to autophagosomes by interacting with LC3 through the LIR [23]. Several such receptors have been discovered, such as SQSTM1/p62 and NBR1 for aggrephagy and OPTN, NDP52, and Tax1BP1 for mitophagy, which can drive autophagic degradation of the corresponding ubiquitinated cargos [23]. Accumulating evidence has demonstrated that ubiquitination can also serve as a signal for pexophagy induction. In the following sections, we discuss the ubiquitin-binding receptors, ubiquitinated substrates, and E3 ligases/deubiquitinases involved in mammalian pexophagy.

### 2.1. Ubiquitin-Binding Receptors in Pexophagy

Attaching a ubiquitin moiety to the cytosolic domains of peroxisomal membrane proteins, such as PMP34 and PMP70, induces autophagic degradation of peroxisomes, suggesting that decorating peroxisomes with artificially ubiquitinated proteins is sufficient to drive pexophagy [24]. The receptor p62 recognizes ubiquitinated peroxisome proteins and delivers the peroxisome to autophagosomes for lysosomal degradation. It was later found that NBR1, another ubiquitin-binding receptor, is also required for pexophagy [25]. Notably, p62 is not required for pexophagy when NBR1 is in excess, but p62 binding to NBR1 significantly increases pexophagy efficiency [25]. These studies were performed with cells grown in normal growth culture, suggesting that NBR1 and p62 can function as the ubiquitin-binding receptors to induce pexophagy under basal conditions. Other studies found that NBR1 and/or p62 are also required for pexophagy induced by oxidative stresses [26,27] and PEX3 overexpression [28], which is discussed below. It remains to be determined whether other mammalian ubiquitin-binding receptors, such as OPTN, NDP52, or Tax1BP1, are involved in pexophagy.

### 2.2. Role of PEX5 Ubiquitination in Pexophagy

**Figure 1:**
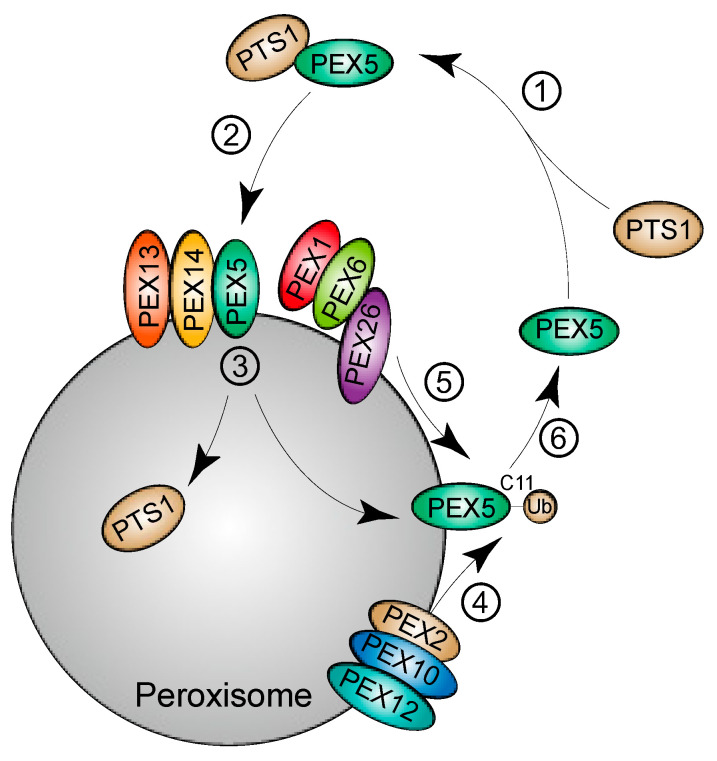
Model for the import of peroxisomal matrix proteins. PTS1-containing cargos are synthesized in the cytoplasm and recognized by the receptor PEX5 (①). PEX5 interacts with the peroxisome-associated docking proteins PEX13 and PEX14 and transports the cargos to the matrix (②). After the release of the cargos (③), PEX5 is recycled back to the cytoplasm for the next round of import (④⑤⑥). Extraction of PEX5 from peroxisomes depends on the monoubiquitination of PEX5 at C11, which is catalyzed by the E3 ligase complex (PEX2, PEX10, and PEX12) (④). The AAA complex (PEX1-PEX6-PEX26) facilitates the extraction process (⑤).

Most peroxisomal matrix proteins possess a peroxisomal targeting signal termed PTS1, consisting of noncleaved C-terminal tripeptide serine-lysine-leucine (SKL) or conserved SKL variants. PEX5 recognizes PTS1-containing cargos and transports them into the peroxisomal matrix. After releasing the cargos, the peroxisome-localized PEX5 proteins are recycled to the cytosol in an ATP-dependent manner for further rounds of import [29]. PEX5 monoubiquitination at the conserved cysteine 11 (C11) facilitates its extraction from peroxisomes by the AAA complex, which consists of PEX1, PEX6, and PEX26 [30]. PEX5 monoubiquitination at C11 is catalyzed by the RING (Really Interesting New Gene) E3 ligase complex, consisted of PEX2, PEX10, and PEX12 (Figure 1). Besides C11, monoubiquitination at K464 by the E3 ligase TRIM37 stabilizes PEX5 and promotes the import of peroxisomal matrix proteins [31]. Both of the monoubiquitination events use members of the UbcH5a/b/c family as the E2 enzymes [31,32]. PEX5 is also polyubiquitinated, which may serve as a quality control mechanism to prevent the accumulation of non-functional PEX5 proteins at peroxisome membranes in response to oxidative stress [29]. In addition to its role as a receptor for matrix protein import, ubiquitinated PEX5 proteins have been reported to serve as substrates for receptor-mediated pexophagy.

**Figure 2:**
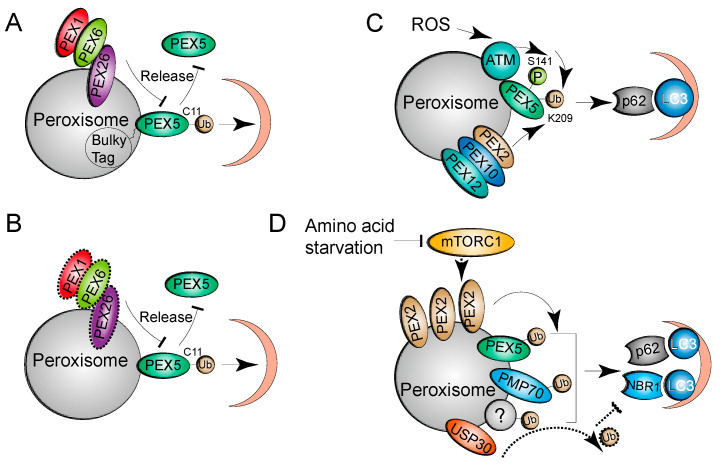
Mechanisms of ubiquitin-dependent pexophagy. (**A**) PEX5 fused to a C-terminal bulky tag can be monoubiquitinated at C11 but fails to be extracted back to the cytosol by the AAA complex. The accumulated monoubiquitination of the PEX5 fusion proteins serves as a signal to direct pexophagy. (**B**) Loss of AAA complex function (dashed border) results in the accumulation of monoubiquitinated PEX5 proteins in peroxisomes, which promotes pexophagy. (**C**) Activation of ATM by ROS phosphorylates PEX5 at S141, which facilitates PEX5 monoubiquitination at K209 by the PEX2, PEX10, and PEX12 complex (E3 ligase complex). The autophagy receptor p62 recognizes ubiquitinated PEX5 and recruits the core autophagy machinery to eliminate damaged peroxisomes in response to oxidative stresses. (**D**) Under conditions of amino acid starvation, PEX2 proteins are stabilized upon mTORC1 inhibition. Upregulation of PEX2 promotes the ubiquitination of peroxisomal proteins, including PEX5, PMP70, and other unknown proteins, which serve as signals for NBR1 and p62-mediated pexophagy. The deubiquitinase USP30 removes the ubiquitin from peroxisomal proteins and inhibits pexophagy during amino acid starvation.

Overexpression of PEX5 proteins fused to a bulky C-terminal tag can trigger pexophagy in SV40 large T antigen-transformed mouse embryonic fibroblasts [33]. The PEX5 fusion proteins are normally monoubiquitinated at C11 but are not released from the peroxisomal membrane, likely because of the bulky tag (Figure 2A). Interestingly, this pexophagy mechanism is cell type-specific and does not depend on NBR1 and p62. Another study showed that pexophagy is induced by the accumulation of ubiquitinated PEX5 proteins on peroxisomal membranes due to the loss of AAA complex function [17] (Figure 2B). Different from the mammalian study, loss of AAA complex function renders peroxisomal import ineffectual and promotes pexophagy independent of the accumulation of ubiquitinated Pex5 proteins in yeast [34]. In addition to the cysteine residue, PEX5 monoubiquitination at Lys 209 (K209) is required for pexophagy in response to ROS [26]. During oxidative stress, ataxia-telangiectasia mutated (ATM) kinase phosphorylates PEX5, promoting PEX5 monoubiquitination at K209. Ubiquitinated PEX5 is recognized by p62 for the subsequent pexophagy process (Figure 2C). Further work is needed to determine whether NBR1 is involved in ROS-driven pexophagy.

As it is also required for peroxisome biogenesis, PEX5 ubiquitination may serve as a quality control mechanism to maintain peroxisome homeostasis. When cells are grown under conditions favoring peroxisome biogenesis, ubiquitinated PEX5 proteins are normally recycled for the next import cycle. However, when cells are grown under conditions not conducive to peroxisome biogenesis, the above studies suggest that PEX5 ubiquitination may accumulate in peroxisomes and serve as a pexophagy signal to remove export-defective or dysfunctional peroxisomes [17,26,33].

Overexpression of the peroxisomal membrane protein PEX3 induces pexophagy with characteristic ubiquitinated and clustered peroxisomes independent of the ubiquitination status of PEX3. The ubiquitinated substrates in pexophagy remain to be identified [28]. Another study showed that multiple peroxisome-localized proteins, including PEX5, PMP70, and some unknown substrates, are ubiquitinated under amino acid starvation conditions, suggesting that PEX5 is not the sole substrate and that ubiquitination of multiple proteins may cooperate to direct starvation-induced pexophagy [35] (Figure 2D). Other ubiquitinated substrates that direct pexophagy remain to be determined.

### 2.3. E3 Ligases/Deubiquitinase in Pexophagy

The overall ubiquitination status of proteins is determined by the coordinated actions of the respective E3 ligases and deubiquitinases. The peroxisomal RING E3 ubiquitin ligase complex, comprising PEX2, PEX10, and PEX12, is critical for the monoubiquitination of PEX5 at C11, which is required for PEX5 receptor recycling [36] (Figure 1). The complex is also required for PEX5 ubiquitination at K209, which is involved in ROS-induced pexophagy [26] (Figure 2C). Intriguingly, under amino acid starvation conditions, PEX2, but not PEX10 or PEX12, acts as the E3 ubiquitin ligase for pexophagy [35]. The study showed that the PEX2 protein level is increased when mTORC1 is inhibited by amino acid starvation or upon treatment with the mTOR inhibitor rapamycin. mTORC1 inhibition may stabilize PEX2 proteins, while its activation may promote proteasomal degradation [35]. After upregulation of PEX2, the ubiquitination of peroxisomes is enhanced, which induces NBR1 and p62-dependent pexophagy [35] (Figure 2D).

USP30 was initially identified as the deubiquitinase to regulate PARK2-mediated mitophagy [37], and later, two independent studies reported that USP30 is required for pexophagy [38,39]; both studies found that a fraction of USP30 localizes to peroxisomes, as well as mitochondria. Sylvie Urbé’s laboratory found that USP30 inhibits basal pexophagy, while Peter Kim’s laboratory found that USP30 overexpression prevents amino acid starvation-induced pexophagy by counteracting the E3 activity of PEX2 (Figure 2D). It is unclear how PEX2 and USP30 cooperate to regulate the overall ubiquitin status of peroxisomes. One possibility is that mTORC1 inhibition leads to inactivated USP30 and activates PEX2, which ensures maximum ubiquitination of peroxisomes for pexophagy induction under amino acid starvation conditions. This hypothesis has not yet been proven. The dual roles of USP30 in mitophagy and pexophagy suggest that peroxisomes and mitochondria may be coordinatively maintained through interplay involving degradation. As discussed above, the E3 ligase PEX2 or the PEX2-PEX10-PEX12 complex play critical roles in peroxisome biogenesis, and the deubiquitinase USP30 is involved in mitophagy in addition to having a role in pexophagy. It remains to be determined whether other specific enzymes are involved in modulating the ubiquitination status of peroxisomes for autophagic degradation.

## 3. Ubiquitin-Independent Pexophagy

Cellular cargo can be delivered for autophagy independently of ubiquitin status, which can be achieved through protein–protein interaction motifs, ubiquitin-like modifiers, and sugar- or lipid-based signaling [13]. For example, FAM134 proteins deliver fragmented ER structures into autophagosomes by directly interacting with LC3/GABARAP proteins through its C-terminal LIR [40]; cardiolipin, a lipid exposed on the outer membrane upon mitochondrial depolarization, serves as a signal for mitophagy [41]. Several regulators have also been found to regulate mammalian pexophagy in a ubiquitin-independent pathway. We discuss these mechanisms in the following sections.

### 3.1. Role of the PEX14-LC3 Interaction in Ubiquitin-Independent Pexophagy

**Figure 3:**
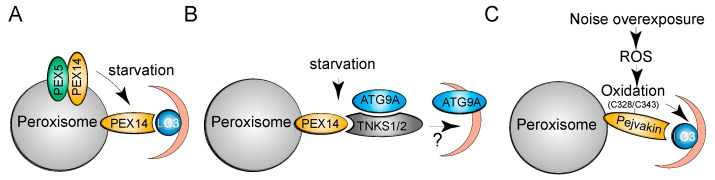
Mechanisms of ubiquitin-independent pexophagy. (**A**,**B**) LC3 outcompetes PEX5 for binding to PEX14 and sequesters peroxisomes into autophagosomes for autophagic degradation under starvation conditions. In parallel, TNKS1/2 proteins associate with PEX14 and ATG9A and promote starvation-induced pexophagy. (**C**) Noise overexposure increases cellular ROS and causes oxidation of pejvakin at C328/C343, which is required for pejvakin-LC3B interaction and pexophagy.

The peroxisomal membrane protein PEX14 is critical for PEX5 docking at peroxisomes and matrix protein import [42] (Figure 1). PEX14 interacts with autophagosome-anchored LC3-II and mediates pexophagy under starvation conditions in CHO cells [43]. Although an LIR was not detected in PEX14, it was found that LC3-II interacts with the transmembrane domain and outcompetes PEX5 for binding to PEX14 [44] (Figure 3A). The mutually exclusive interaction may serve as a quality control mechanism to regulate peroxisome abundance; the PEX14-PEX5 interaction ensures the normal progression of peroxisomal import, whereas binding of free PEX14 to LC3-II triggers autophagy machinery to drive pexophagy.

### 3.2. Role of the TNKS1/2-PEX14 Interaction in Ubiquitin-Independent Pexophagy

Tankyrase 1 (TNKS1) and tankyrase 2 (TNKS2) belong to the poly (ADP-ribose) polymerase family. An interacting proteomic analysis revealed that TNKS1/2 associate with PEX14 and localize to peroxisomes [45]. Overexpression of TNKS1/2 induces pexophagy under basal conditions, and their depletion prevents amino acid starvation-induced pexophagy, independently of their ADP-ribose transferring activity. Peroxisome-localized TNKS1/2 proteins may promote pexophagy by associating with ATG9A (Figure 3B). ATG9-containing vesicles interact transiently with phagophores and deliver additional membranes to growing autophagosomes [46]. Further work is needed to examine how the PEX14-TNKS1/2-ATG9A interaction recruits autophagosomes and induces pexophagy. As TNKS1/2 are not ubiquitin-binding proteins and no interaction between TNKS1/2 and NBR1 or p62 has been detected, the study suggests that TNKS1/2 mediate pexophagy in a ubiquitin-independent manner [45].

### 3.3. Role of Pejvakin in Ubiquitin-Independent Pexophagy

Pejvakin belongs to the gasdermin protein family and has a markedly different structure than other gasdermin protein members [47]. Noise overexposure increases ROS levels and causes oxidative damage to auditory hair cells. A study showed that pejvakin recruits LC3 proteins through a defined LIR motif and triggers the autophagic degradation of noise-induced oxidative stress-damaged peroxisomes [48] (Figure 3C). Pejvakin has no ubiquitin-binding domain and does not interact with NBR1 or p62, suggesting that pejvakin mediates pexophagy in a ubiquitin-independent manner. Importantly, it was found that the two cysteine residues in pejvakin, C328/C343, are essential for ROS-induced pejvakin-LC3B interaction and pexophagy (Figure 3C). As cysteine oxidation can relax the compact conformation and increase the binding capacity of proteins [49], it is likely that pejvakin depends on ROS-induced cysteine oxidation and acts as a ROS sensor to mediate pexophagy.

## 4. Mammalian Pexophagy Receptors

To define a protein as a receptor for selective autophagy, it must play at least two roles: recognizing the specific cargos and recruiting the autophagy machinery for the lysosomal degradation of cargos. Yeast pexophagy receptors have been identified: Atg30 in Komagataella phaffii and Atg36 in S. cerevisiae. The two receptors do not share amino acid sequence homology but act similarly in targeting peroxisomes for degradation. Both receptors localize to peroxisomes, and their overexpression induces pexophagy even under conditions that normally promote peroxisome proliferation. They localize at peroxisomal membranes and recruit the core autophagy machinery to peroxisomes by interacting with scaffold proteins (Atg11 and Atg17) and the ubiquitin-like protein Atg8 [21].

Homologs of Atg30 and Atg36 have not been found in mammalian cells. As discussed above, several regulators, such as NBR1, p62, PEX14, TNKS1/2, and Pejvakin, have been proposed as pexophagy receptors. In addition, a study showed that acyl-CoA binding domain-containing protein 5 (ACBD5), a human ortholog of yeast Atg37, localizes to peroxisomes and may function as a pexophagy receptor [50]. Compared to the well-established receptor role for Atg30/Atg36 in yeast, the precise mechanisms of how these receptor candidates regulate pexophagy are unclear and remain to be further investigated. In addition, the roles of these proteins are not restricted to pexophagy. For example, NBR1 and p62 are known to act as ubiquitin-binding receptors for several other selective autophagy, such as aggrephagy and xenophagy [13]. Specific receptors recognize a particular cargo, such as those receptors involved in mitophagy (BNIP3, BNIP3 L, and FUNDC1) [51]. Hence, other specific pexophagy receptors remain to be identified. Finally, these receptor candidates are identified in different cell types. It remains to be determined whether they are cell type-specific receptors and/or can cooperate with others. As the receptor is the basis of selective autophagy, further exploration of known and unknown receptors remains a major challenge in the mammalian pexophagy field.

## 5. Pexophagy-Inducing Conditions

By using HaloTag technology to examine peroxisome dynamics, it was found that peroxisomes in cultured mammalian cells have a half-life of 1.5~2 days under basal growth conditions and treatment with 3-methyladenine, an inhibitor of the class III phosphoinositide 3-kinase (PI3K) complex, prevents the degradation of peroxisomes, suggesting that autophagy is involved in peroxisome turnover [7]. In addition to basal turnover, pexophagy can be triggered by various stimuli, as described below.

### 5.1. Pexophagy Induced by the Discontinuation of Phthalate Ester Treatment

The proliferation of peroxisomes can be induced by a group of hypolipidemic drugs [9] and other chemicals [10,11]. These peroxisome proliferators may stimulate the activity of PPAR-α and its downstream effectors, which increase the size and number of peroxisomes, as well as the level of the enzymes involved in fatty acid metabolism [52,53]. Following induction of peroxisomes by a 2-week treatment with the phthalate ester DEHP (a PPAR-α agonist) in mouse livers, degradation of peroxisomes can be induced within 1 week after discontinuation of the DEHP induction. However, this rapid removal was strikingly impaired in the livers of ATG7-deficient mice [15,16], suggesting that autophagy is essential for the selective clearance of excess peroxisomes. This study provides a good induction model with which to study pexophagy in vivo. However, a similar strategy failed to induce pexophagy in several cultured cell lines [16]. This may reflect the complexity of pexophagy induction with these chemicals in the mouse model.

### 5.2. Pexophagy Induced by Modulated Activities of Peroxisome Biogenesis Factors

A subset of genes, including PEX genes, dynamin-related protein 1 (DRP1), and mitochondrial fission factor, are involved in peroxisome biogenesis. Peroxisome deficiency caused by loss of these PEX gene functions may serve as a pexophagy signal triggering the removal of deficient peroxisomes. As discussed above, loss of AAA complex function induces pexophagy [17]. Modulating the activity of other PEX proteins has also been found to regulate pexophagy, such as those found for PEX3 [28] and PEX14 [42]. DRP1-mediated fission promotes mitophagy by fitting elongated mitochondria into nascent autophagosomes or sequestering damaged subdomains from the healthy mitochondrial network [54,55]. Since peroxisomes share several division proteins with mitochondria, such as DRP1 [56], and fission is important for yeast pexophagy [57], it remains to be determined whether modulating the activity of fission proteins can affect mammalian pexophagy.

### 5.3. Pexophagy Induced by Oxidative Stress

Peroxisomes are sites of ROS generation and decomposition and play crucial roles in maintaining cellular oxidative homeostasis. Oxidative stress disrupts the redox environment required for normal peroxisome function and would induce pexophagy. Catalase, a major peroxisomal matrix protein, catalyzes the breakdown of H_2_O_2_ within peroxisomes. Its inhibition elevates ROS levels and can induce NBR1-dependent pexophagy in nutrient-depleted cell cultures [58]. ROS-mediated pexophagy is also observed in the liver tissue of catalase-knockout mice subjected to prolonged fasting [59]. Notably, pexophagy induced by catalase inhibition does not occur in basal cell culture or fully fed mice, suggesting a close relationship between starvation and ROS-mediated pexophagy. In addition, ROS elevation caused by the addition of H_2_O_2_ [26], treatment with the chemical 1,10-phenanthroline (Phen) [60], or loss of the heat shock protein HSP9 [27] induces significant pexophagy. Mechanistically, oxidative stress may induce PEX5 ubiquitination-dependent pexophagy by activating ATM signaling, as discussed above [26] (Figure 2C).

### 5.4. Pexophagy Induced by Hypoxia

Oxygen (O_2_) signaling regulates the homeostasis of peroxisomes, as oxidative metabolism in peroxisomes requires available O_2_. As crucial transcription factors involved in O_2_ signaling, HIF-1/2α activate a plethora of genes in response to hypoxia [61]. The stability of HIF-1/2α subunits is regulated by the von-Hippel-Lindau (VHL) protein. VHL ubiquitinates HIF-1/2α for proteasomal degradation under normoxia, while hypoxia or loss of VHL function results in the stabilization of HIF-1/2α subunits and activation of hypoxia signaling [61].

A study showed that loss of VHL induces autophagic degradation of peroxisomes in mouse livers. Deletion of HIF-2α, but not HIF-1α, ablates the induction of peroxisome degradation, suggesting that HIF-2α mediates pexophagy. The study showed that the autophagy receptors NBR1 and SQSTM1 localize to peroxisomes and are degraded by pexophagy, suggesting that NBR1 and p62 may be involved in HIF-2α-mediated pexophagy. The study provides evidence of hypoxia signaling in regulating peroxisome homeostasis through pexophagy [62]. HIF-2α might induce the expression of an E3 ubiquitin ligase that increases the ubiquitination status of peroxisomes and the subsequent recruitment of the receptors NBR1 and p62 [63]. Further work is needed to investigate the underlying mechanisms of HIF-2α-mediated pexophagy.

### 5.5. Pexophagy Induced by Amino Acid Depletion

Amino acids are not only essential materials for protein synthesis but are also crucial energy and carbon sources utilized by many other metabolic pathways. Amino acids activate signaling by mTORC1, a master regulator of cell growth [64]. As discussed above, several studies have shown that amino acid starvation inhibits mTORC1 signaling and can induce pexophagy, suggesting that mTORC1 signaling can maintain the abundance of peroxisomes by inhibiting the pexophagy process. Different pathways have been found for starvation-induced pexophagy, such as PEX2 upregulation and PEX14- or TNKS1/2-mediated pathways [35,43,45]. However, the direct targets downstream of mTORC1 that regulate pexophagy remain to be identified.

As discussed above, several stimuli can trigger pexophagy in mammalian cells and have improved our understanding of pexophagy. Several considerations need to be taken into account when using these stimuli to induce pexophagy. First, these stimuli can cause profound cellular stress and have many pexophagy-independent effects. For examples, oxidative stress, hypoxia, and starvation all induce general autophagy, which also reduces the peroxisome contents; they may also reduce the number and size of peroxisomes through inhibiting the biogenesis process. It needs to be determined whether the observed reduction in peroxisomes is caused directly by pexophagy or indirectly by other pathways. Second, these pexophagy-inducing conditions are performed in different cells or mouse models. Different types of proteins (the receptors, etc.) mediate the induction of pexophagy in different yeast model systems. Future work is needed to discriminate these mechanisms of pexophagy in response to different stimuli in mammalian cells. Third, these stimuli reduce peroxisomes to a lesser extent in mammals than in yeast. This difference may indicate a quality control mechanism ensuring that only dysfunctional or incompetent peroxisomes are degraded and the remaining peroxisomes attenuate the stress induced by these stimuli. As several hundred to a thousand peroxisomes are present in mammalian cells [65], a reduction in only a fraction of peroxisomes upon stimulation can make pexophagy difficult to quantify. Hence, caution must be used when monitoring pexophagy induced by these stimuli. Finding a stimulus that can dramatically reduce the peroxisome number would benefit pexophagy studies.

## 6. Roles of Pexophagy in Health and Disease

Peroxisomes are essential cellular organelles, and aberrant functions have been implicated not only in PBDs but also in many other human diseases, such as cancer, neurodegenerative disorders, aging, and diabetes [1]. Aberrant regulation of pexophagy can disrupt peroxisome homeostasis, thereby causing human diseases. We discuss the roles of pexophagy in several human diseases below.

### 6.1. Role of Pexophagy in PBDs

A study showed that loss of the AAA complex does not inhibit the import of matrix proteins but triggers NBR1-dependent pexophagy [17]. In the study, autophagy inhibition rescues peroxisome number, protein import, and function in PEX1^G843D^ (the most common PBD mutation) patient fibroblasts [17]. Another study showed that overexpression of USP30 inhibits pexophagy by reducing the ubiquitination of peroxisomes and can rescue peroxisome loss in PEX1^G843D^ patient fibroblasts [39]. As mutations in AAA complex genes are the most common among PBD patients, these studies suggest that pexophagy is critical for the majority of the peroxisomes lost in PBD patients. Currently, there is no curative therapy for PBDs; these studies suggest an exciting therapeutic opportunity for PBD patients by targeting pexophagy. The recently developed PEX1^G844D^ transgenic mouse recapitulates many features of PBDs and can serve as a good model to test the effectiveness of these targeted therapies in vivo [18,66].

However, these studies were mainly performed by using the PEX1^G843D^ mutant but not the full deletion. PEX1^G843D^ mutant can achieve an estimated 15% complementation activity [67]. The residual activity may improve the peroxisomal functions when these cells are treated with autophagy inhibitors. In yeast, specific inhibition of PEX1 deletion-induced pexophagy does not restore peroxisomal matrix protein import or the peroxisomal function in β-oxidation [68]. Intriguingly, another study found that inhibition of pexophagy with autophagy inhibitors fails to improve peroxisomal functions in PBD cells harboring the PEX1^G843D^ mutation [69]. The authors argue that the different assays used to monitor peroxisomal functions may result in discrepancies in their conclusions [69].

### 6.2. Role of Pexophagy in Cancer

Loss of VHL function is detected in as many as 90% of sporadic human clear cell renal cell carcinomas (ccRCCs), and HIF-2α is known to be a ccRCC driver oncoprotein [70]. The finding that HIF-2α drives pexophagy is in agreement with the detection of high HIF-2α levels and loss of peroxisomes in ccRCC patient samples. As the accumulation of neutral lipids and glycogen are characteristic features of ccRCCs, loss of peroxisomal function through HIF-2α-mediated pexophagy can result in the alteration of lipid metabolism and may contribute to the malignant phenotype. High fructose consumption and metabolism contribute to the development of many pathologic conditions such as cancer [71]. A study found that activation of HIF-2α signaling or loss of peroxisomal function suppresses the expression of the rate-limiting enzyme Ketohexokinase (KHK) and inhibits fructose metabolism [72]. However, the suppression of KHK is not dependent on HIF-2α-induced pexophagy, as recuse of the pexophagy by autophagy inhibition does not restore the expression of KHK [72]. Recent evidence has revealed that the levels of peroxisome proteins or enzymatic activities are either increased or reduced in various cancer types, suggesting that peroxisomes may have a tumor-promoting or tumor-suppressing function [73,74]. Understanding the mechanisms of pexophagy in the control of peroxisome homeostasis is of great importance to decipher the role of peroxisomes in carcinogenesis.

### 6.3. Role of Pexophagy in Neurodegenerative Disease

Neurodegenerative diseases, including Alzheimer’s disease (AD) and Parkinson’s disease (PD), have been known to exhibit increased oxidative stress. Disrupted redox balance can result in neurodegenerative diseases [75]. HSPA9 expression is decreased in the brain tissue of PD patients, and several genetic variants of HSPA9 have been identified in PD patients [76]. A study showed that HSPA9 depletion induces pexophagy by increasing peroxisomal ROS in neuroblastoma cells [27]. Importantly, the expression of wild-type HSPA9, but not PD HSPA9-mutant proteins, rescued the loss of peroxisomes in HSPA9-depleted cells. This study suggested that loss of peroxisomal functions due to aberrant induction of pexophagy may contribute to PD progression. Further investigation is needed to elucidate the mechanism of HSPA9 depletion-induced pexophagy and its pathogenic role in PD.

### 6.4. Role of Pexophagy in Hearing Loss

The increase in ROS by noise exposure causes hearing loss through oxidative damage to auditory hair cells and neurons [77]. Mutation of the pejvakin gene causes nonsyndromic, prelingual, and sensorineural hearing impairment [78]. Pejvakin was found to localize in peroxisomes and is required for the sound-induced proliferation of peroxisomes [79]. In response to sound exposure, pejvakin acts as a ROS sensor and recruits the autophagosome-associated LC3B protein to trigger pexophagy [48]. Restoring pexophagy by expressing pejvakin and LC3B proteins in pejvakin-deficient cells promotes the proliferation of peroxisomes, suggesting that pexophagy precedes and promotes peroxisome proliferation after sound exposure. These studies revealed that pexophagy plays a major role in redox homeostasis and protects auditory hair cells against oxidative damage. Intriguingly, it remains to be examined whether hearing loss commonly found in PBD patients is caused by defective pejvakin-mediated pexophagy.

### 6.5. Role of Pexophagy in HIV-1 Infection

HIV-1 infection is characterized by a progressive decline in the number of CD4^+^ T lymphocytes, ultimately leading to acquired immunodeficiency syndrome in untreated patients. A study showed that expression of HIV viral envelope glycoproteins (Env) triggers massive macroautophagy/autophagy as well as pexophagy, which results in the elimination of mature peroxisomes in CD4^+^ T cells. In addition, Env expression induces a dramatic increase in cellular ROS, which induces cell death [80]. The study suggested that reducing the number of functional peroxisome through pexophagy may enhance oxidative stress, which can damage CD4^+^ T cells and contribute to the acquired immunodeficiency observed in HIV-1-infected patients.

## 7. Concluding Remarks

As discussed above, pexophagy plays a fundamental role in maintaining peroxisome homeostasis in mammalian cells, and aberrant regulation causes or accompanies the progression of many human diseases. Substantial progress has been made in the mammalian pexophagy field; however, numerous questions remain to be explored. A few examples are as follows: (1) The precise mechanisms of identified and unidentified pexophagy receptors/regulators need to be further investigated. (2) It remains to be determined whether the results obtained from the cell cultures can be validated in vivo. (3) It remains to be determined how pexophagy is regulated to adjust to changing environmental conditions. Future work investigating these questions will help us better understand the mechanisms and functions of pexophagy in human health and diseases.

## Data Availability

This statement is excluded as the study did not report any data.
